# Anti-peptidylarginine deiminase-4 antibodies at mucosal sites can activate peptidylarginine deiminase-4 enzyme activity in rheumatoid arthritis

**DOI:** 10.1186/s13075-021-02528-5

**Published:** 2021-06-06

**Authors:** M. Kristen Demoruelle, Hong Wang, Ryan L. Davis, Ashley Visser, Johnny Hoang, Jill M. Norris, V. Michael Holers, Kevin D. Deane, Erika Darrah

**Affiliations:** 1 Division of Rheumatology, University of Colorado Anschutz Medical Campus, 1775 Aurora Court, Mail Stop B-115, Aurora, CO 80045 USA; 2 Division of Rheumatology, The Johns Hopkins University, 5200 Eastern Ave. Suite 5300, Baltimore, MD 21224 USA; 3 Colorado School of Public Health, Aurora, CO USA

**Keywords:** Rheumatoid arthritis, Anti-PAD4 antibodies, Lung, Sputum, Saliva

## Abstract

**Background:**

Mucosal sites are hypothesized to play a role in the development of rheumatoid arthritis (RA). Since serum anti-peptidylarginine deiminase (PAD)4 antibodies, including a subset that cross-react with PAD3 (PAD3/4), are specific for RA and associate with severe disease, we sought to examine whether anti-PAD4 and anti-PAD3/4 antibodies were present in the lung and oral mucosa of subjects with RA and “at-risk” for RA.

**Methods:**

We included 37 RA, 25 healthy control, and 46 subjects “at-risk” for RA based on familial RA and/or serum anti-citrullinated protein antibody (ACPA) positivity. Paired serum, sputum, and saliva were evaluated for anti-PAD4 and anti-PAD3/4 using immunoprecipitation and ACPA using ELISA. Immunoglobulins (Ig) were purified from representative samples, and their effect on citrullination of histone H3 by recombinant human PAD4 was measured by anti-citH3 immunoblot.

**Results:**

Anti-PAD4 antibodies were detected in the serum of 6/37 (16.2%), sputum of 3/37 (8.1%), and saliva of 3/33 (9.1%) RA subjects and in the serum and sputum of 1/46 (2.2%) at-risk subjects. None of the healthy controls had anti-PAD4 antibodies at any site. Serum, sputum, and salivary anti-PAD4 antibodies were more prevalent in RA subjects with RA duration >2 years. Purified antibodies from representative anti-PAD4-positive and anti-PAD3/4-positive sputum were primarily of the IgA isotype and able to increase PAD4 enzymatic activity.

**Conclusions:**

Anti-PAD4 antibodies are present in the sputum and saliva of a portion of RA patients and are infrequent in at-risk subjects. Importantly, the ability of anti-PAD4, and particularly anti-PAD3/4, antibodies in the sputum to enhance PAD4 enzymatic activity suggests that anti-PAD4 may play an active role in the RA lung.

## Background

Rheumatoid arthritis (RA) is a systemic autoimmune disease characterized by disease-specific autoantibodies, a hallmark of which is anti-citrullinated protein antibodies (ACPAs). Citrullinated proteins are generated via the deimination of arginine residues by the calcium-dependent peptidylarginine deiminase (PAD) enzymes. There are five PAD isoenzymes in humans (PAD1, PAD2, PAD3, PAD4, and PAD6) [1], with PAD2 and PAD4 most strongly implicated in RA pathogenesis. Autoantibodies to PAD4 have been identified in the blood of 23–45% of chronic RA patients and in 17–22% of patients with early disease (<2 years) [2–8]. Anti-PAD4 antibodies also associate with ACPA and more severe joint disease, suggesting a role in pathogenesis. A subset of anti-PAD4 antibodies cross-reacts with the related enzyme PAD3 (termed anti-PAD3/4 antibodies) and have been associated with the most severe joint damage and imaging evidence of interstitial lung disease (ILD) [3]. Anti-PAD3/4 antibodies possess the unique ability to markedly enhance PAD4 enzyme activity at physiologic calcium concentrations, which can lead to increased protein citrullination.

Anti-PAD4 antibodies have also been identified in the serum of 18% of subjects during the pre-clinical phase of RA development, prior to the onset of inflammatory arthritis [9]. Data suggest that inflammation and antibody generation in the pre-clinical phase of RA may initially develop at mucosal sites, such as the lung and oral mucosae [10–12]. These data include our previous findings that ACPAs are generated in the sputum of subjects with established RA, as well as RA-free subjects who are “at-risk” of developing RA in the future [13, 14]. Mucosal involvement has also been implicated in the pathogenesis of RA after joint disease onset [15, 16]. Our prior finding that serum anti-PAD3/4 antibodies are associated with imaging evidence of lung disease in established RA [3] suggests that these antibodies may be present and active in the lung. However, their presence at mucosal sites, including the lung, has not been evaluated. As such, we sought to explore the presence of anti-PAD4 and anti-PAD3/4 antibodies in the sputum and saliva of RA patients, as well as subjects at-risk for RA, and define their effect on PAD4 enzyme activity.

## Methods

### Study subjects

Subjects were recruited from the Studies of the Etiology of RA Lung Study that is described in detail elsewhere [13, 14]. Briefly, this lung study was designed to study the biomarkers of autoimmunity in the lung during different phases of RA development. For this cross-sectional study, stored samples were included from RA subjects (*N* = 37) and healthy controls (*N* = 25). RA subjects were all serum ACPA-positive based on anti-cyclic citrullinated peptide (CCP) ELISA testing (QUANTA Lite CCP3.1 IgG/IgA or CCP3 IgG, Inova Diagnostics, San Diego, CA, USA) and had a medical chart review to confirm RA diagnosis by 1987 RA classification criteria and 2010 RA classification criteria or previously diagnosed with anti-CCP+ RA by a board-certified rheumatologist. Healthy controls had no personal or family history of RA and were serum anti-CCP-negative. We also included 46 subjects at-risk for RA defined as having a first-degree relative with RA (*N* = 20), having serum anti-CCP positivity identified through community or clinical screening (*N* = 15), or having both (*N* = 11). At-risk subjects had no clinical or historical evidence of inflammatory arthritis at the time of sample collection.

### Study visit

All subjects had a paired collection of blood and sputum. The majority (79%) also had saliva collected prior to sputum collection. Standardized questionnaires were used to obtain demographic information and self-reported histories of smoking and chronic lung disease.

### *HLA-DRB1* testing

Immunogenetic analysis was performed on DNA isolated from the whole blood of each patient to determine the presence of *HLA-DRB1* alleles containing the shared epitope using previously described methodologies [17].

### Sputum and saliva collection and processing

Induced sputum was collected using inhaled hypertonic saline and established protocols that have been previously described [13, 14]. A portion of subjects (33 RA, 21 controls, and 31 at-risk) provided an unstimulated saliva sample prior to sputum induction. Saliva samples with volume remaining after anti-PAD4 antibody testing were also tested for ACPA, and this included 28 RA, 19 controls, and 28 at-risk subjects (see below for a description of antibody testing methodologies). All samples were stored at −80°C.

### Serum, sputum, and saliva ACPA testing

Paired serum, sputum, and saliva were tested for ACPA using anti-CCP3.1 (IgG/IgA, Inova Diagnostics, San Diego, CA, USA) ELISA. In serum, the cutoff level for anti-CCP3.1 positivity established by the manufacturer was used. For sputum, a cutoff level for anti-CCP3.1 positivity was set at the 95th percentile of sputum anti-CCP3.1 levels in a separate healthy control group (N = 100, median age 37 years, 71% female, 22% ever smokers). For saliva, a cutoff level for anti-CCP3.1 positivity was set at the 95th percentile of salivary anti-CCP3.1 levels in a separate healthy control group (N = 80, median age 48 years, 60% female, 24% ever smokers).

### Serum, sputum, and saliva anti-PAD testing

Anti-PAD4 and anti-PAD3/4 antibodies were tested using an established two-tiered quantitative immunoprecipitation method [2]. Briefly, subject sera (1 μl), sputum (10 μl), or saliva (20 μl) were incubated with 1 μl of ^35^*S*-methionine-labeled PAD4 or PAD3 generated via in vitro transcription and translation (Promega) for 1 h at 4°C. Radiolabeled immune complexes were immunoprecipitated with 40 μl Protein A beads and washed, and bound antigen was eluted using 2× sodium dodecyl sulfate buffer. The immunoprecipitated proteins were separated by gel electrophoresis, visualized by radiography, and quantified using densitometry. Densitometry values were background-corrected and normalized to a known positive reference serum analyzed in parallel. A normalized value of >0.140 anti-PAD arbitrary units was considered positive for either anti-PAD4 or anti-PAD3 antibodies based on the analysis of known negative samples. Samples that were negative for reactivity to both PADs were considered “anti-PAD negative”; those that were positive for PAD4 reactivity but negative for PAD3 were considered “anti-PAD4 mono-reactive,” and those that were positive for both PAD4 and PAD3 reactivity were defined as “anti-PAD3/4 cross-reactive,” based on our previous work [2]. Isotype-specific immunoprecipitation was also performed, using a similar protocol as described above, for all samples using anti-IgA, IgM, or IgG-coupled agarose beads (Sigma; cat#A3316, A2691, and A9935, respectively) to define the proportion of anti-PAD4 or anti-PAD3/4 antibodies of each isotype present.

### PAD4 activity testing

To determine the effect of autoantibodies on PAD4 enzymatic activity, IgG and IgA were co-purified from anti-PAD-negative, anti-PAD4 mono-reactive, and anti-PAD3/4 cross-reactive serum and sputum using an equal mixture of Protein A/G agarose (Pierce; cat#20423) and Peptide M agarose (InvivoGen; cat# gel-pdm-2) beads. This mixture purifies predominantly all four IgG subclasses and IgA isotypes, with minimal purification of IgM. The concentration of total Ig was determined by NanoDrop (Thermo), >95% purity confirmed by Coomassie stain, and composition confirmed by immunoblot with goat anti-human IgG antibody and rabbit anti-human IgA antibody (Jackson Laboratories). The effect of purified Ig on PAD4 activity was evaluated using recombinant human PAD4, purified in-house as previously described [18]. PAD4 (10 nM) was pre-incubated with 1 μM purified Ig for 45 min at 4°C, followed by incubation with 700 μM histone H3 substrate for 3 h at 37°C at increasing calcium chloride concentrations (i.e., 0.2 and 2 mM), as previously described [2]. Citrullination of histone H3 was evaluated by anti-citrullinated histone H3 immunoblotting (ab5103, Abcam).

### Statistical analysis

Subject characteristics were compared between the groups using Kruskal-Wallis testing for age and chi-square/Fisher’s exact test for dichotomous variables, including the prevalence of anti-PAD antibody positivity. All analyses were performed using the SPSS software, version 25, and figures were generated using GraphPad Prism version 8.

## Results

### Demographics

Subject demographics are listed in Table 1. Subjects were predominately female and non-Hispanic white. Healthy controls were younger and less likely to be current smokers. RA subjects were more likely to have at least one *HLA-DRB1* shared epitope allele. Only 9/37 (24%) RA subjects had chronic RA, defined as having been diagnosed with RA more than 2 years ago.

**Table 1:** Subject characteristics

	RA (*N* = 37)	At-risk (*N* = 46)	Controls (*N* = 25)	*p* value
Age, median (IQR)	56 (42–61)	52 (46–63)	36 (28–54)	<0.01
Female	27 (73)	29 (63)	21 (84)	0.18
Non-Hispanic white	22 (60)	32 (70)	18 (72)	0.51
Ever-smoker	17 (46)	18 (39)	8 (32)	0.54
Current smoker	7 (19)	5 (11)	0 (0)	0.05
≥1 shared epitope allele^1^	23 (72)^1^	20 (44)	13 (52)	0.04
Chronic lung disease^2^	9 (24)	8 (17)	4 (16)	0.86
RA duration >2 years	9 (24)	–	–	–
Serum anti-CCP+	37 (100)	26 (57)	0 (0)	<0.01
Serum anti-PAD4+	6 (16)	1 (2)	0 (0)	0.02
Serum anti-PAD3/4+	2 (5)	1 (2)	0 (0)	0.60
Sputum anti-CCP+	18 (49)	11 (24)	0 (0)	<0.01
Sputum anti-PAD4+	3 (8)	1 (2)	0 (0)	0.28
Sputum anti-PAD3/4+	1 (3)	1 (2)	0 (0)	1.0
Salivary anti-CCP+^3^	11 (30)	4 (14)	0 (0)	<0.01
Salivary anti-PAD4+^3^	3 (9)	0 (0)	0 (0)	0.11
Salivary anti-PAD3/4+	0 (0)	0 (0)	0 (0)	1.0

Values are listed as *N* (%) unless otherwise noted

^1^Only 32 of 37 RA subjects had DNA available for SE testing

^2^Chronic lung disease was defined as a self-report of a health care provider diagnosis of chronic asthma, emphysema, bronchitis, bronchiectasis, interstitial lung disease, or other chronic lung diseases

^3^Only 33 RA, 31 at-risk, and 21 controls had saliva available for testing. After anti-PAD4 testing, only 28 RA, 28 at-risk, and 19 controls had saliva available for anti-CCP testing

### Prevalence of serum, sputum, and salivary anti-PAD4 antibodies

**Fig. 1:**
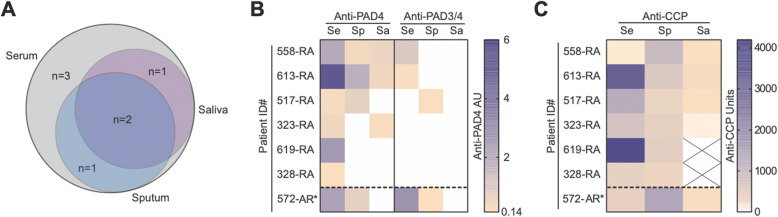
Detection of anti-PAD4 and anti-PAD3/4 IgG at different anatomical sites. Antibodies to PAD4, including those of the PAD3/4 subset, were detected in paired serum (Se), sputum (Sp), and saliva (Sa) samples from RA patients (*n* = 37), individuals at-risk (AR) for RA (*n* = 46), and healthy controls (*n* = 35). **a** The overlap in anti-PAD4 antibody positivity at the three anatomical sites in anti-PAD4-positive individuals (*n* = 7) is depicted using a Venn diagram. Anti-PAD3/4 positivity did not overlap between serum, sputum, and saliva. Heat maps show the anti-PAD antibody arbitrary units (AU) **b** and anti-CCP units **c** in anti-PAD-positive individuals (*n* = 7) at the various anatomical sites. Salivary samples that were not available for anti-CCP testing are marked with an X

We found that 6/37 (16.2%) RA subjects had serum anti-PAD4 IgG, and 2 of those 6 subjects (33.3%) possessed the PAD3/4 antibody subset in the serum (Fig. 1 and Table 1). In addition, we found anti-PAD4 IgG in the sputum of 3/37 (8.1%) RA subjects, and 1 of those 3 (33.3%) had sputum anti-PAD3/4 antibodies. In the subset of subjects with saliva available, we found that 3/33 (9.1%) RA subjects had salivary anti-PAD4 IgG, but none was anti-PAD3/4-positive. All patients with sputum or saliva anti-PAD4 antibodies were also positive for anti-PAD4 antibodies in their serum, and 2/3 had anti-PAD4 antibodies in both their sputum and saliva. As such, 1/37 (2.7%) RA subjects had sputum anti-PAD4 positivity in the absence of salivary anti-PAD4 positivity, and 1/37 (2.7%) RA subjects had salivary anti-PAD4 antibody in the absence of sputum positivity.

We also found serum and concomitant sputum anti-PAD4 and anti-PAD3/4 IgG positivity in 1/46 (2.2%) at-risk subjects (Fig. 1 and Table 1). Anti-PAD4 was not detected in the saliva of any at-risk subjects. While statistical comparisons between the groups were not made based on having only a single positive at-risk subject, the serum and sputum anti-PAD4-positive at-risk subject was a 65-year-old woman, never smoker with no history of chronic lung disease, and had serum and sputum anti-CCP antibodies but not salivary anti-CCP antibodies. This at-risk subject was seen in a follow-up research study visit 21 months later and had not developed RA. Importantly, neither anti-PAD4 nor anti-PAD3/4 antibodies were detected in the serum, sputum, or saliva of any of the healthy controls tested.

In patients with established RA, serum, sputum, and salivary anti-PAD4 IgG demonstrated a non-significant trend toward an association with sputum anti-CCP positivity (Table 2). Both RA subjects with serum anti-PAD3/4 antibodies, including the subject with sputum anti-PAD3/4 positivity, were sputum anti-CCP-positive. There was no association between serum, sputum, and salivary anti-PAD4 IgG and salivary anti-CCP positivity (data not shown). Serum anti-PAD4 positivity was associated with chronic RA (Table 2), and there was a non-significant trend toward a similar association between sputum and salivary anti-PAD4 positivity and chronic RA. There was no significant difference in age, sex, smoking history, shared epitope positivity, or history of chronic lung disease between serum, sputum, or salivary anti-PAD4-positive and anti-PAD-negative RA subjects. Of note, 3 RA subjects reported a history of ILD, of which 1/3 (33%) was positive for anti-PAD4 antibodies in the serum, sputum, and saliva. This individual also had the anti-PAD3/4 antibody subset in their serum but not sputum or saliva.

**Table 2:** Factors associated with serum, sputum, and salivary anti-PAD4 IgG in RA subjects

	Serum PAD4+ (*N* = 6)	Serum PAD4− (*N* = 31)	*p* value	Sputum PAD4+ (*N* = 3)	Sputum PAD4− (*N* = 34)	*p* value	Saliva PAD4+ (*N* = 3)	Saliva PAD− (*N* = 30)	*p* value
Age, median (IQR)	51 (29−64)	56 (42−60)	0.71	43 (26−59)	57 (42−61)	0.27	43 (30−59)	55 (41−61)	0.33
Female	5 (83)	22 (71)	1.0	2 (67)	25 (74)	1.0	2 (67)	23 (77)	1.0
Non-Hispanic white	5 (83)	17 (55)	0.37	2 (67)	20 (59)	1.0	3 (100)	18 (60)	0.28
Ever-smoker	1 (17)	16 (52)	0.19	1 (33)	16 (47)	1.0	1 (33)	13 (43)	1.0
Current smoker	1 (17)	6 (19)	1.0	1 (33)	6 (17)	0.48	1 (33)	5 (17)	0.46
≥1 shared epitope allele^1^	4 (80)	19 (70)	1.0	3 (100)	20 (69)	0.54	2 (100)	19 (70)	1.0
Chronic lung disease^2^	2 (33)	7 (23)	0.62	1 (33)	8 (24)	1.0	1 (33)	7 (23)	1.0
RA duration >2 years	4 (67)	5 (16)	0.02	2 (67)	7 (21)	0.14	2 (67)	7 (23)	0.17
Sputum anti-CCP+	5 (83)	13 (42)	0.09	3 (100)	15 (44)	0.11	3 (100)	13 (43)	0.10
Sputum anti-CCP level, median (IQR)	450 (325−753)	231 (101−603)	0.09	664 (342−1020)	275 (114−507)	0.12	664 (426−1020)	252 (114−634)	0.10

Values are listed as *N* (%) unless otherwise noted

^1^Only 32 of 37 RA subjects had DNA available for SE testing

^2^Chronic lung disease was defined as a self-report of a health care provider diagnosis of chronic asthma, emphysema, bronchitis, bronchiectasis, interstitial lung disease, or other chronic lung diseases

### Serum, sputum, and salivary anti-PAD4 antibody isotypes and effect on PAD4 activity

**Fig. 2:**
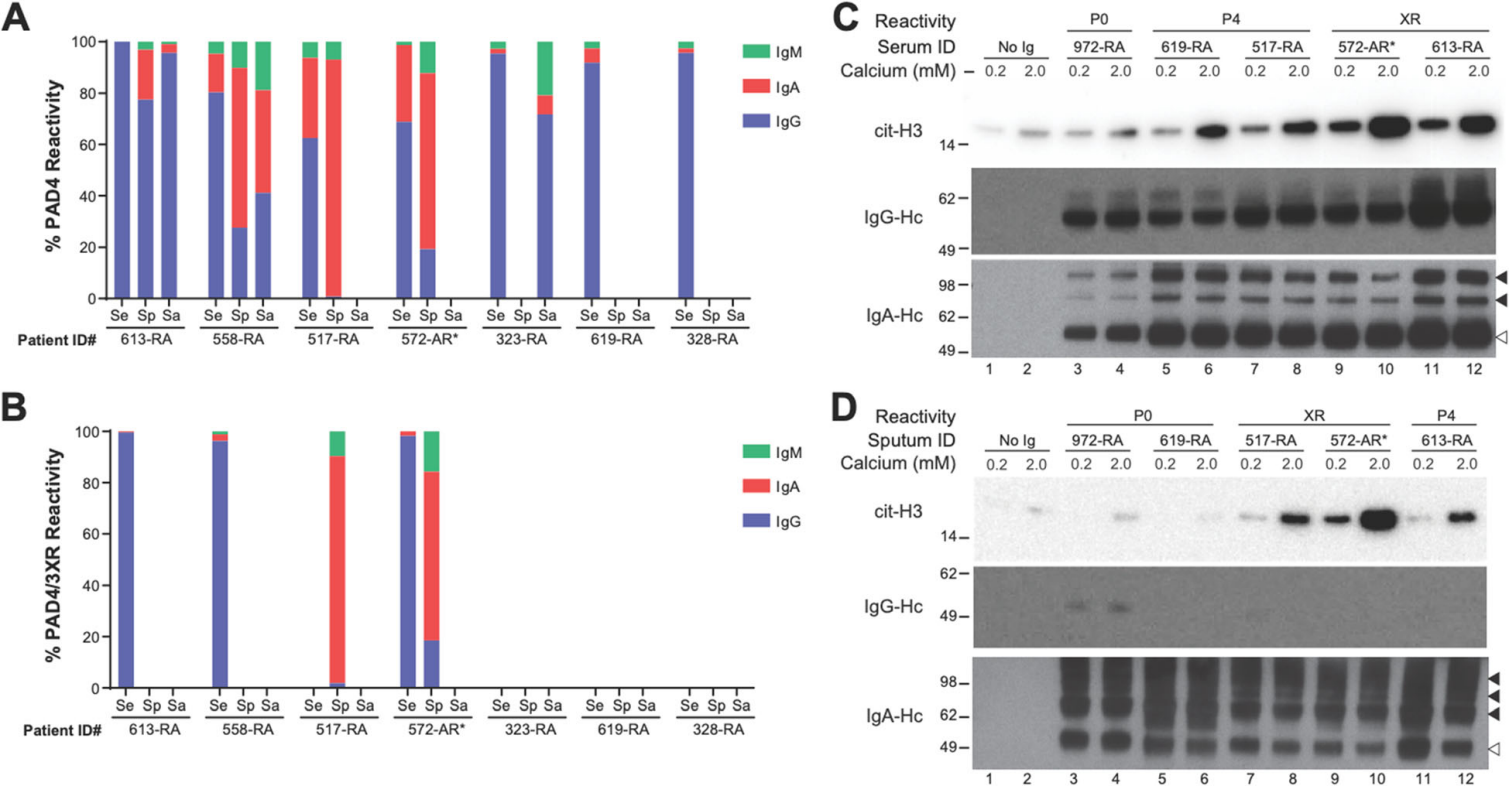
Analysis of anti-PAD4 antibody isotypes and ability to activate PAD4. **a**, **b** IgG- (blue), IgA- (red), and IgM-specific (green) immunoprecipitations were performed on paired serum (Se), sputum (Sp), and saliva (Sa) of individuals with anti-PAD4 antibodies (n = 7) to define the isotypes of anti-PAD4 **a** and the anti-PAD3/4 subset **b** at each anatomic site. The total reactivity to PAD4 or PAD3/4 was calculated, and the % reactivity of each antibody isotype is shown. **c**, **d** No IgG or purified total IgG and IgA from serum **c** or sputum **d** from anti-PAD-negative (P0), anti-PAD4 mono-reactive (P4), or anti-PAD3/4 cross-reactive (XR) RA or at-risk (AR*) samples was pre-incubated with PAD4, prior to incubation with histone H3 substrate and either 0.2 or 2.0mM calcium at 37°C. Immunoblotting was performed to detect citrullination of histone H3 (cit-H3), human IgG (heavy chain; IgG-Hc), and human IgA (heavy chain; IgA-Hc). Monomeric IgA (open arrowheads) and multimeric IgA (closed arrowheads) are indicated

The discovery of anti-PAD4 IgG in sputum and saliva prompted us to define the isotype distribution of anti-PAD4 antibodies present at these different anatomical sites (Fig. 2a, b). As expected, anti-PAD4 and anti-PAD3/4 antibodies in the serum were predominately of the IgG isotype. Sputum, on the other hand, was enriched for anti-PAD4 and anti-PAD3/4 cross-reactive antibodies of the IgA isotype, in most patients. Interestingly, IgG was the predominant isotype of anti-PAD4 antibodies in the saliva, with intermediate amounts of IgA and significantly less IgM observed.

Serum anti-PAD4 IgG from some patients with RA has been shown to modulate the catalytic activity of PAD4 [3, 19], with a subset of patients possessing IgG with the capacity to augment the ability of PAD4 to citrullinate substrates. We previously found that serum IgG from anti-PAD3/4-positive patients was the most agonistic [2]. To determine whether the agonistic activity of anti-PAD4 and anti-PAD3/4 antibodies differed based on anatomical site, IgG and IgA were co-purified from paired serum and sputum samples from representative anti-PAD-negative, anti-PAD4 mono-reactive, and anti-PAD3/4 cross-reactive RA or at-risk patient samples and tested for their ability to modulate PAD4 activity in vitro (Fig. 2c, d). Since PAD4-activating antibodies were previously shown to reduce the amount of calcium required for catalysis [2], two different physiologic calcium concentrations (0.2 and 2.0mM) were tested. Despite normalizing to total Ig, the relative abundance of IgG and IgA in the serum and sputum varied between individuals. However, regardless of the anatomical source of Ig (serum or sputum), anti-PAD3/4 Ig (Fig. 2c, lanes 9–12; and Fig. 2d, lanes 7–10), and to a lesser extent anti-PAD4 mono-specific Ig (Fig. 2c, lanes 5–8; Fig. 2d, lanes 11–12), was able to enhance the catalytic activity of PAD4 to citrullinate histone H3 over anti-PAD-negative Ig and no Ig controls (Fig. 2c, lanes 1–4; Fig. 2d, lanes 1–6).

## Discussion

We found anti-PAD4 antibodies in the sputum and saliva of a portion of patients with established RA and characterized the isotype and functional capacity of these autoantibodies. Similar to serum anti-PAD4 antibodies, sputum and salivary anti-PAD4 antibodies were associated with a longer duration of RA (>2 years) as well as demonstrated a trend toward an association with anti-CCP positivity in the lung. However, unlike serum and salivary anti-PAD4 antibodies, which were primarily of the IgG isotype, sputum anti-PAD4 autoantibodies were predominantly IgA. While the overall prevalence of sputum and salivary anti-PAD4 antibodies was low in our cohort, they were highly specific for RA, when compared to at-risk and control subjects (98–100% specific). In addition, sputum anti-PAD3/4 antibodies, which were predominately IgA, robustly enhanced the catalytic activity of PAD4, suggesting they could amplify PAD4 activity directly in the lung in a subset of patients with RA.

Our current study fills important gaps in the published literature and highlights potentially important differences in the temporality and role of mucosal sites in the development of ACPA and the anti-PAD4 autoantibody responses. Data support that ACPAs likely originate at mucosal sites and mucosal ACPA generation is highly prevalent in established RA. These data include the findings that ACPAs are present in the sputum of a subset of at-risk individuals in the absence of serum ACPA positivity and that ACPAs are highly prevalent in the sputum and bronchoalveolar lavage fluid of established RA patients [13, 14, 20]. In contrast, our study revealed that anti-PAD4 antibodies are a rare finding in the serum and sputum of at-risk subjects and are only observed in the sputum of a subset of established RA patients with serum anti-PAD4 positivity. Furthermore, serum ACPA positivity is high in the pre-clinical phase of RA while our current study and published data demonstrate that serum anti-PAD4 antibodies are much less frequent in the pre-clinical phase and are associated with longer RA disease duration [4, 9, 21]. In aggregate, these data suggest that anti-PAD4 antibodies likely follow a different model of origin compared to ACPA with anti-PAD4 antibodies likely a later event in the evolution of RA.

Additional support for anti-PAD4 antibodies being amplifiers of disease, rather than causes of ACPA-positive RA, comes from the discovery of serum anti-PAD3/4 antibodies that are associated with disease duration and severe joint disease in RA patients [2]. These antibodies have the capacity to augment PAD4 enzymatic activity suggesting that they may drive the continued production of citrullinated autoantigens at sites of inflammation. Our finding of sputum anti-PAD3/4 IgA in three patients with established RA suggests that these antibodies may drive continued citrullinated antigen generation in the lung. Further studies in larger cohorts are needed to define the clinical significance of sputum anti-PAD4 and anti-PAD3/4 antibodies and whether they may play a direct role in amplifying inflammation in the lung of a subset of at-risk and RA individuals. Part of this must be to define the precise contribution of anti-PAD4 IgG and IgA to effector functions in the lung, outside of PAD4 enzyme activation, including Fc receptor engagement and cellular effects. It is also important to consider the prevalence and role of antibodies targeting the related enzyme PAD2 in future sputum studies, since serum anti-PAD2 antibodies have been found in a serologically and genetically distinct group of RA patients with milder joint and lung disease [22].

Serum anti-PAD3/4 antibodies have been associated with radiographic evidence of ILD in RA patients [3], even in the absence of symptoms. Despite the small sample size in our study, it is interesting to note that only one of the three RA subjects with sputum anti-PAD4 antibodies had a prior clinical diagnosis of ILD. In addition, we have previously reported that serum anti-PAD4 antibodies were not associated with a history of smoking [23]. This is supported in this current study, in which serum, sputum, and salivary anti-PAD4 antibodies were found to develop in several individuals without a history of smoking. Our study was not designed to examine sputum anti-PAD4 antibodies in RA-related lung diseases, but our confirmation in this study that these antibodies can be present in the lung supports future studies that can specifically address whether sputum anti-PAD4 or anti-PAD3/4 antibodies are associated with the prevalence, severity, or future development of RA-related lung diseases.

Caveats to our study include the low prevalence of serum anti-PAD4 antibody positivity in this cohort of RA patients compared to previous reports (16.2% vs. 23–45%, respectively) [4, 5, 7]. It is likely that the low prevalence of anti-PAD4 in our study is due to the low percentage of RA patients (24%) who had chronic RA. In published studies, serum anti-PAD4 antibody prevalence is lower in early RA, and in our study, serum anti-PAD4 antibodies were present in 4/9 (44%) chronic RA patients. Our study also had a low prevalence of sputum and salivary anti-PAD4 positivity, which limits our ability to identify all possible associations between clinical characteristics and sputum or salivary anti-PAD4 antibodies in RA patients. In this study, we found also a trend toward an association of anti-PAD4 and anti-CCP antibodies in the sputum. While we cannot confirm the citrulline specificity of these sputum antibodies, our published work has demonstrated the presence of citrulline-specific ACPA in the sputum of RA patients [24]. In addition, the cross-sectional nature of the at-risk cohort limits the inferences about the evolution of anti-PAD4 autoantibodies in these patients. We also cannot completely exclude the possibility of antibody translocation from the serum to the lung or oral mucosae in this study. However, it is unlikely given that the predominate anti-PAD4 antibody isotype in the serum was IgG, but was predominantly IgA in sputum, suggesting local production of IgA in the lung mucosa. In addition, regardless of the source (i.e., local generation or translocation from the serum), we demonstrated that the anti-PAD4 antibodies in the sputum of some RA patients have the capacity to activate PAD4, suggesting that they may contribute to the local production of citrullinated proteins directly in the lung. Importantly, our novel finding of anti-PAD4 and anti-PAD3/4 antibodies at mucosal sites strongly supports the need for future studies to characterize the prevalence and clinical features associated with this antibody subset in RA.

## Conclusions

In conclusion, we identified sputum anti-PAD4 and anti-PAD3/4 antibodies in a portion of RA subjects and one subject at-risk for RA, and salivary anti-PAD4 antibodies in a subset of patients with established disease. These data support the long-standing hypothesis that mucosal sites may play an important role in the pathogenesis of RA.

## Data Availability

The datasets used and/or analyzed during the current study are available from the corresponding authors on reasonable request.

## Abbreviations

ACPAs: Anti-citrullinated protein antibodies
CCP: Cyclic citrullinated peptide
ILD: Interstitial lung disease
Ig: Immunoglobulins
PAD: Peptidylarginine deiminase
RA: Rheumatoid arthritis
